# Supplementary material for: Analysis of associated malformations by computed tomography in adults with polysplenia syndrome: A pilot study
Source: PLoS One. 2024 Dec 20;19(12):e0312548. doi: 10.1371/journal.pone.0312548 (PMC11661641; doi:10.1371/journal.pone.0312548)
Supplement: Attachment — Submitted filename: PONE-D-24-28190_reviewer.docx [file pone.0312548.s001.docx]

PLOS ONE

**Analysis of associated malformations in adults with polysplenia syndrome on CT**

--Manuscript Draft--

| **Manuscript Number:** | PONE-D-24-28190 |
| --- | --- |
| **Article Type:** | Research Article |
| **Full Title:** | Analysis of associated malformations in adults with polysplenia syndrome on CT |
| **Short Title:** | Analysis of associated malformations in adults with polysplenia syndrome on CT |
| **Corresponding Author:** | kefu liu  The Affiliated Suzhou Hospital of Nanjing Medical University Suzhou, Jiangsu CHINA |
| **Keywords:** | Polysplenia syndrome; Adult; Computed tomography |
| **Abstract:** | Objective: To summarize the associated malformations of polysplenia syndrome (PS) in adults by CT.  Methods: The incidence of malformations associated with polysplenia syndrome (PS) in twelve adult patients was retrospectively analyzed by CT imaging.  Results: The number of splenic nodules ranged from three to twelve; the splenic nodules were located in the left upper quadrant in nine patients and in the right upper quadrant in three patients. A short pancreas was present in all twelve patients. Midgut malrotation was present in eight patients. Situs inversus totalis was present in two patients. In nine patients, the hepatic segmental inferior vena cava was agenesis, with the hepatic vein directly converging into the right atrium, and continuation of the azygos vein was found. The preduodenal portal vein was present in six patients. Left lung heterotaxy was found in nine patients. The inferior vena cava was bilateral in one patient. Aberrant right subclavian arteries, bilateral common carotid arteries sharing trunks, abnormal renal vein branching and routing, and abdominal portal vein branching were also found in individual patients.  Conclusion: PS is a complex malformation syndrome involving multiple systems. The most common malformations are short pancreas, and other malformations, such as left lung heterogeneity, hepatic segmental IVC agenesis with continuation of the azygos vein, midgut malrotation, preduodenal portal vein, and left atrial heterotaxy, have relatively high prevalence rates. |
| **Order of Authors:** | Shuangshuang Xu |
|  | Jinghua Chen |
|  | Xiaoqin Jiang |
|  | Ping Xie |
|  | Xiang Fang |
|  | Yan Gao |
|  | Jian Huang |
|  | kefu liu |
| **Additional Information:** |  |
| **Question** | **Response** |
| **Financial Disclosure**  Enter a financial disclosure statement that describes the sources of funding for the work included in this submission. Review the [submission guidelines](http://journals.plos.org/plosone/s/submission-guidelines#loc-financial-disclosure-statement) for detailed | The author(s) received no specific funding for this work. |

| requirements. View published research articles from [*PLOS ONE*](https://journals.plos.org/plosone/) for specific examples.  This statement is required for submission and **will appear in the published article** if the submission is accepted. Please make sure it is accurate. |  |
| --- | --- |
| **Funded studies**  Enter a statement with the following details:   - Initials of the authors who received each award - Grant numbers awarded to each author - The full name of each funder - URL of each funder website - Did the sponsors or funders play any role in the study design, data collection and analysis, decision to publish, or preparation of the manuscript? |  |
| Did you receive funding for this work? |  |
| **Competing Interests**  Use the instructions below to enter a competing interest statement for this submission. On behalf of all authors, disclose any [competing interests](http://journals.plos.org/plosone/s/competing-interests) that could be perceived to bias this work—acknowledging all financial support and any other relevant financial or non- financial competing interests.  This statement is required for submission and **will appear in the published article** if the submission is accepted. Please make sure it is accurate and that any funding sources listed in your Funding Information later in the submission form are also declared in your Financial Disclosure statement.  View published research articles from  [*PLOS ONE*](http://journals.plos.org/plosone/) for specific examples. | The authors have declared that no competing interests exist. |

| **NO authors have competing interests**  Enter: *The authors have declared that no competing interests exist.*  **Authors with competing interests**  Enter competing interest details beginning with this statement:  *I have read the journal's policy and the authors of this manuscript have the following competing interests: [insert competing interests here]* |  |
| --- | --- |
| * typeset |  |
| **Ethics Statement**  Enter an ethics statement for this submission. This statement is required if the study involved:   - Human participants - Human specimens or tissue - Vertebrate animals or cephalopods - Vertebrate embryos or tissues - Field research   Write "N/A" if the submission does not require an ethics statement.  General guidance is provided below. Consult the [submission guidelines](http://journals.plos.org/plosone/s/submission-guidelines#loc-guidelines-for-specific-study-types) for detailed instructions. **Make sure that all information entered here is included in the Methods section of the manuscript.** | This retrospective study was approved by the institutional review board (No. K2023177, The Affiliated Suzhou Hospital of Nanjing Medical University) with a waiver of written informed consent. |

| **Format for specific study types**  **Human Subject Research (involving human participants and/or tissue)**   - Give the name of the institutional review board or ethics committee that approved the study - Include the approval number and/or a statement indicating approval of this research - Indicate the form of consent obtained (written/oral) or the reason that consent was not obtained (e.g. the data were analyzed anonymously)   **Animal Research (involving vertebrate animals, embryos or tissues)**   - Provide the name of the Institutional Animal Care and Use Committee (IACUC) or other relevant ethics board that reviewed the study protocol, and indicate whether they approved this research or granted a formal waiver of ethical approval - Include an approval number if one was obtained - If the study involved *non-human primates*, add *additional details* about animal welfare and steps taken to ameliorate suffering - If anesthesia, euthanasia, or any kind of animal sacrifice is part of the study, include briefly which substances and/or methods were applied   **Field Research**  Include the following details if this study involves the collection of plant, animal, or other materials from a natural setting:   - Field permit number - Name of the institution or relevant body that granted permission |  |
| --- | --- |
|  |  |
| **Data Availability**  Authors are required to make all data underlying the findings described fully available, without restriction, and from the time of publication. PLOS allows rare exceptions to address legal and ethical concerns. See the [PLOS Data Policy](http://journals.plos.org/plosone/s/data-availability) and [FAQ](http://journals.plos.org/plosone/s/data-availability#loc-faqs-for-data-policy) for detailed information. | No - some restrictions will apply |

| A Data Availability Statement describing where the data can be found is required at submission. Your answers to this question constitute the Data Availability Statement and **will be published in the article**, if accepted. |  |
| --- | --- |
| **Important:** Stating ‘data available on request from the author’ is not sufficient. If your data are only available upon request, select ‘No’ for the first question and explain your exceptional situation in the text box. |  |
| Do the authors confirm that all data underlying the findings described in their manuscript are fully available without restriction? |  |
| **Describe where the data may be found in full sentences. If you are copying our sample text, replace any instances of XXX with the appropriate details.**   - If the data are **held or will be held in a public repository**, include URLs, accession numbers or DOIs. If this information will only be available after acceptance, indicate this by ticking the box below. For example: *All XXX files are available from the XXX database (accession number(s) XXX, XXX.*). - If the data are all contained **within the manuscript and/or Supporting Information files**, enter the following: *All relevant data are within the manuscript and its Supporting Information files.* - If neither of these applies but you are able to provide **details of access elsewhere**, with or without limitations, please do so. For example:   *Data cannot be shared publicly because of [XXX]. Data are available from the XXX Institutional Data Access / Ethics Committee (contact via XXX) for researchers who meet the criteria for access to confidential data.*  *The data underlying the results presented in the study are available from (include the name of the third party* | All relevant data are within the manuscript and its Supporting Information files. |

| *and contact information or URL).*   - This text is appropriate if the data are owned by a third party and authors do not have permission to share the data.   * typeset |  |
| --- | --- |
| Additional data availability information: | Tick here if the URLs/accession numbers/DOIs will be available only after acceptance of the manuscript for publication so that we can ensure their inclusion before publication. |

Manuscript	[Click here to access/download;Manuscript;text .docx](https://www2.cloud.editorialmanager.com/pone/download.aspx?id=36958378&guid=4e77e2c0-7d67-4741-ad75-4225a12392bc&scheme=1)

**Title:** Analysis of associated malformations in adults with polysplenia syndrome on CT

# Authorship:

Xu Shuangshuang^1^, Chen Jinghua^2^, Jiang Xiaoqin^3^, Xie Ping^3^, Fang Xiang^4^, Gao Yan^5^, Huang Jian^2^, Liu Kefu^3^

# Affiliations and addresses:

1. Department of Radiology, Suzhou Lili Hospital, Suzhou, Jiangsu, China
2. Department of Radiology, Taicang City Hospital of Traditional Chinese Medicine, Taicang, Jiangsu, China;
3. Department of Medical Imaging, The Affiliated Suzhou Hospital of Nanjing Medical University, Gusu School of Nanjing Medical University, Suzhou, Jiangsu, China
4. Department of Radiology, The First Affiliated Hospital of Soochow University, Suzhou, Jiangsu, China
5. Department of Radiology, The Wuxi Xishan people’s hospital, Wuxi, Jiangsu, China

# Correspondence to:

Kefu Liu, MD, PhD, Department of radiology, The Affiliated Suzhou Hospital of Nanjing Medical University, Suzhou Municipal Hospital, Gusu School, Nanjing Medical University, No.242, GuangJi Road, Suzhou, Jiangsu, 215008, China. Tel: 86- 0512-62363306.E-mail address: [lkf77@126.com.](mailto:lkf77@126.com)

# # Contributed equally to this paper

Xu Shuangshuang, Chen Jinghua, Jiang Xiaoqin, Xie Ping

# Author conflicts of interest

The authors declare no conflict of interest, financial or otherwise.

# Funding

1. Suzhou key diseases project (LCZX202212)
2. Suzhou Science and Technology Bureau Project (SKY2023064)
3. Suzhou Science and Technology Bureau Project (SKYD2023255)

# Analysis of associated malformations in adults with polysplenia syndrome on CT

**Abstract**

*Objective:* To summarize the associated malformations of polysplenia syndrome (PS) in adults by CT.

*Methods:* The incidence of malformations associated with polysplenia syndrome (PS) in twelve adult patients was retrospectively analyzed by CT imaging.

*Results:* The number of splenic nodules ranged from three to twelve; the splenic nodules were located in the left upper quadrant in nine patients and in the right upper quadrant in three patients. A short pancreas was present in all twelve patients. Midgut malrotation was present in eight patients. Situs inversus totalis was present in two patients. In nine patients, the hepatic segmental inferior vena cava was agenesis, with the hepatic vein directly converging into the right atrium, and continuation of the azygos vein was found. The preduodenal portal vein was present in six patients. Left lung heterotaxy was found in nine patients. The inferior vena cava was bilateral in one patient. Aberrant right subclavian arteries, bilateral common carotid arteries sharing trunks, abnormal renal vein branching and routing, and abdominal portal vein branching were also found in individual patients.

*Conclusion:* PS is a complex malformation syndrome involving multiple systems. The most common malformations are short pancreas, and other malformations, such as left lung heterogeneity, hepatic segmental IVC agenesis with continuation of the azygos vein, midgut malrotation, preduodenal portal vein, and left atrial heterotaxy, have relatively high prevalence rates.

*Key words:* Polysplenia syndrome; Adult; Computed tomography

# 1. Introduction

Polysplenia syndrome (PS), which is a rare syndrome of congenital anomalous spleen development with other multisystem malformations ^1^, is also known as left atrial isomerism syndrome (LAIS) because of the frequent combination of left atrial isomerism ^2^. PS occurring in adults is very rare and has been reported mostly in case studies ^3-5^. Compared with other examinations, thoracic and abdominal multilayer computed tomography (CT) is the best means to clarify the details of PS combined with various anatomical variants ^6^; therefore, in this study, we retrospectively analyzed the CT imaging data of twelve adults of PS to summarize the concomitant deformities in adults of PS with the aim of improving the understanding of concomitant deformities of PS, avoiding misdiagnosis of anatomical variants as lesions and causing unnecessary surgical injuries.

# 2. Materials and Methods

2.1 *Patients*

This retrospective study was approved by the institutional review board (No. 2023177, The Affiliated Suzhou Hospital of Nanjing Medical University) with a waiver of written informed consent.

CT images of 12 PS patients from January 2015 to March 2024 were retrospectively collected. The data for this study were accessed from 10/10/2023 to10/10/2024. There were 5 males and 7 females, with an age range of 30-74 years. All 12 patients underwent plain CT examination of the chest and abdomen, and CT enhancement was performed for 6 patients.

2.2 *Analysis methods*

Two radiologists with 7 and 20 years of experience in diagnostic imaging observed and analyzed the CT images of each patient with PS, respectively, and the concomitant deformities of the PS were recorded.

# 3. Results

3.1 *Medical history*

A history of cholangiocarcinoma with cirrhosis, cholangiocarcinoma in the porta hepatis with cholecystitis, chronic inflammation of the bile ducts and duodenum, biliary stones, arrhythmia and right kidney stones, left kidney stones, polyps of the colon, and atrial septal defects were recorded in one patient each, and the other patient did not have any special medical history.

3.2 *CT imaging findings*

The concomitant deformities of the PS of 12 patients shown on CT are shown in Table 1.

Respiratory system: Nine patients showed left lung heterogeneity (i.e., both lungs and bilateral bronchi showed left-sided morphology) (Figure 1a).

Digestive system: Splenic: The number of splenic nodules ranged from three to twelve, with sizes ranging from 3×4×6 mm to 18×30×88 mm (Figure 1b). Polysplenia nodules were located in the left upper quadrant in nine patients and in the right upper quadrant in three patients. Splenic nodules were distributed along the external and posterior parts of the greater curvature of the stomach (Figure 1c).

Pancreas: A short pancreas was observed in all twelve patients (the head and uncinate process of the pancreas were enlarged, and the body and tail of the pancreas were short or absent) (Figure 1b).

Hepatobiliary system: Six patients exhibited normal performance. The liver and gallbladder were found in the left upper quadrant in two patients. A small left lobe of the liver was detected in one patient, a horizontal liver with a right gallbladder was detected in two patients (Figure 1d), and a horizontal liver and median gallbladder were detected in one patient (Figure 1e).

Gastrointestinal tract: The stomach was located on the right side (Figure 1c) in three patients, and midgut malrotation was found in eight patients (Figure 1f).

3.3 *Cardiovascular system*

Heart: Dextrocardia was found in two patients (Figure 1g).

Atrium: CT suggested possible left atrial heterotaxy in five patients (Figure 1h).

Aortic arch: The right aortic arch (RAA) and dextral pulmonary trunk were affected in two patients (Figure 1i).

Superior vena cava (SVC): A persistent left superior vena cava (PLSVC) was found in one patient.

Inferior vena cava (IVC): The absence of the hepatic segment of the IVC (Figure 1j and 1k), which continued upward as a thickened azygos vein were found in nine patients (Figure 1k), and a bilateral IVC was found in one patient (Figure 1f).

Portal vein: The preduodenal portal vein (PDPV) was present in six patients (Figure 1l); in one patient, the portal vein traveled in front of the head of the pancreas and then bifurcated into two branches; the thicker branch traveled along the anterior border of the liver to the diaphragmatic surface of the liver into the liver, and the thinner branch entered the liver in the first hepatic portal (Figure 1m).

Hepatic vein: In nine patients, the hepatic vein exited the second porta hepatis and converged directly into the right atrium (Figure 1k).

Renal veins: Two patients had unilateral renal veins with double-branched variants: one patient had both renal veins converging directly into the azygos vein (Figure 1n), one patient had the left renal vein traveling posterior to the abdominal aorta (Figure 1o), and one patient had the right renal vein flowing back into the azygos vein and injecting upward into the SVC. In two patients, the left renal vein first converged into the hemiazygos vein and then into the azygos vein in the posterior mediastinum.

Common carotid artery (CCA): Bilateral CCA shared trunks in two patients (Figure 1p).

Others: An aberrant right subclavian artery (ARSA) was found in two patients (Figure 1p).

# 4. Discussion

4.1 *Pathogenesis and survival rates*

PS was first reported by Baillie in 1788^7^, but the exact cause of PS is still unclear, and studies have shown that its occurrence is closely related to embryonic development, inheritance, teratogenic factors, genetic mutations and other factors^6^. The 5th-7th week of embryo development is a critical stage for the development and rotation of the spleen, atrial septum, trunk of the conus artery and atrioventricular valve, as well as the process of the gastrointestinal tract returning to the abdominal cavity from the umbilical cord for rotation. Cardiovascular and visceral anomalies occur when this process is impaired. PS is very rare, with an infantile morbidity rate of only 1/250000, and the degree of congenital heart disease malformations determines the survival of patients with PS. The mortality rate in the first year is 50-60%; 25% of patients with PS survive to the age of 5 years, and only 10% have normal hearts or only minor heart defects and therefore survive to adulthood ^8-10^. Some studies have reported^4,^ ^8^ that the prevalence of PS is

greater in females than in males, and our findings are consistent with the literature.

4.2 *CT findings*
    4.2.1 Multiple spleens

The number of spleens reported in the literature is between two and sixteen ^9^, with different sizes, no main spleen, no increase in the total weight of the spleen, no hypersplenism, and the spleen can be located in the left upper quadrant, right upper quadrant, or both sides of the abdomen, with the left upper quadrant being the most common. The spleen is usually located on the side of the greater curvature of the stomach and shows "blotchy" enhancement in the arterial phase and uniform enhancement in the venous and delayed phase. The degree of enhancement was consistent with that of a normal spleen ^6,^ ^10^.

The number of spleens in this group ranged from three to twelve, twelve of which were located next to the greater curvature of the stomach, three of which were located in the right upper quadrant. Six patients who completed the CT enhancement examination showed arterial "blotchy" and venous uniform enhancement, twelve patients had no main spleen, and the size of the splenic nodules varied, which was consistent with previous reports.

4.2.2 Visceral abnormalities

In our group, all twelve patients had short pancreases, including two patients with pancreatic inversion, which is the most prevalent extrasplenic abnormality. Since the normal pancreas is formed by the fusion of ventral and dorsal pancreatic buds, the ventral pancreatic buds give rise to the uncinate process and head, while the dorsal pancreatic buds give rise to the body and tail, and the development of the dorsal pancreatic buds and the dorsal spleen occurs dorsal to the gastric mesentery ^11^. When the pancreas is dorsally underdeveloped, there is caudal dysplasia of the body and tail of the pancreas, which mostly manifests as a short pancreas. Some of the literature^11-13^ has reported that pancreatic abnormalities are associated with PS, and a few studies^14^ have reported pancreatic malrotation.

In this group, one patient had a small left lobe of the liver accompanied by a right gallbladder, two patients had total visceral inversion manifested as a left liver and gallbladder, two patients had a horizontal liver with a right gallbladder, and one patient had a horizontal liver with a middle gallbladder. Some studies have also reported these hepatobiliary anomalies ^4,^ ^12^.

In our group, there were eight cases of midgut malrotation (8/12, 67%), including two cases of gastrointestinal anteversion (total visceral anteversion), which is consistent with the published literature showing small bowel malrotation in 60.4% of PS patients

4, 15.

In our group, we found kidney stones in two of our twelve patients, and no other genitourinary anomalies, such as double ureter or renal hypoplasia ^16,^ ^17^, were observed.

4.2.3 *Vascular malformations*

In our group, hepatic segmental IVC agenesis with continuation of azygos was found in nine patients. When the hepatic segment of the IVC fails to anastomose with the suprarenal segment during embryonic development, the renal segment and lower

segment will flow back into the SVC via the azygos or hemiazygos vein ^6^, which is considered to be the most common vascular malformation in PS ^8,^ ^18,^ ^19^.

In our group, PDPV was present in six patients. PDPV ^20,^ ^21^ is a rare congenital anomaly, especially in adults, in which the portal vein passes through the anterior part of the duodenum rather than the posterior part and leaves an abnormal vein in the anterior part of the duodenum due to an anomaly of rotation during intestinal rotation or an anomaly of closure of the vitelline vein (umbilical vein), which results in the formation of a portal vein. In patients who require surgery, failure to detect the presence of PDPV before surgery may lead to serious complications such as intraoperative bleeding. In addition, it has been found ^22^ that PDPV may compress the duodenum and biliary tract, causing intestinal obstruction and biliary obstruction.

Renal vein anomalies mainly manifest as retro-aortic renal vein and the right renal vein flowing back into the azygos vein to inject upward into the SVC, and the left renal vein converges into the hemiazygos vein, which converges into the azygos vein in the posterior mediastinum ^4^. Among them, the retro-aortic renal vein usually occurs unilaterally on the left side, which can lead to hematuria, left-sided pain, and a variety of other symptoms, often suggesting posterior nutcracker syndrome (PNCS) ^23^, which is relatively rare and is consistent with the presentation in one patient in our group.

Other vascular malformations, including ARSA and bilateral CCA shared trunks ^4^, were found in our group except vertebral artery origin anomaly. In our study, two special manifestations were not observed in the previous PS-related literatures, which were the bilateral IVC and portal vein abnormalities bifurcated into two branches with the thicker branch traveling along the anterior border of the liver to the diaphragmatic surface of the liver and the thinner branch entering the liver at the first hepatic hilar.

4.2.4 *Lung anomalies*

Nine patients in our group exhibited left lung heterogeneity, with a higher incidence than that in previous studies, which reported an incidence of 55% ^9^. A study ^6^ also revealed that the incidence of bilateral lung heterogeneity on the left side was much lower in adults with PS, which suggested that since adults with PS usually do not have cardiac anomalies such as abnormal pulmonary venous return or ASD, they do not experience bilateral left-sided changes. In our group, there were only two patients with cardiac abnormalities, which is not consistent with previous reports in the literature ^6,^ ^9^. It is hypothesized that the reason for this may be that left lung heterogeneity is related not only to cardiac abnormalities but also to other factors that need further confirmation.

4.2.5 *Cardiac abnormalities*

In this group, five CT scans suggested the possibility of left atrial heterotaxy, one had a history of VSD, and cardiac ultrasound suggested a tumor in the membranous portion of the interventricular septum. PS in adults is usually not combined with cardiac developmental abnormalities or shows mild cardiac abnormalities ^3^. These abnormalities mainly include positional abnormalities of the heart, such as dextrocardia^8^; structural abnormalities, such as ASD, ventricular septal defect, right ventricular double outlet, atrial septal hypertrophy, and lipoma^10^; and arrhythmias, such

as atrioventricular block and bradycardia^24^, some of which are difficult to diagnose via CT and usually rely on cardiac ultrasound and electrocardiogram.

4.2.6 *Comorbid tumors*

In our group, there were two patients with cholangiocellular carcinoma, which was also reported in the past ^25^, and the cause is hypothesized to be related to biliary obstruction due to developmental malformations of the biliopancreatic duct and recurrent infections of the biliary system in patients with PS. Previous case reports ^14,^ ^25-27^ have shown that PS can be combined with malignant tumors, such as hepatocellular carcinoma, intrahepatic cholangiocarcinoma, gastric carcinoma, rectal carcinoma, and breast cancer. However, there is no evidence that PS is a precancerous syndrome or that there is a greater incidence of tumors than in the normal population.

4.2.7 *Differential diagnosis*

PS needs to be differentiated from asplenia syndrome (AS), accessory spleen, simple visceral inversion, enlarged lymph nodes in the abdominal cavity, and other nodular masses in the abdominal cavity ^7,^ ^8,^ ^28^. (1) AS can present as congenital splenic hypoplasia or absence of the spleen, with trilobarism of both lungs (right-sided heterotaxy of both lungs), a prevalence of median liver, and more frequent and more severe cardiovascular malformation than PS, and the IVC is located on the same side of the spine as the aorta. (2) Accessory spleen: There is a large primary spleen, with a large difference in volume between the primary and secondary spleens, whereas there is no primary spleen in the PS. (3) Simple inversion of the viscera: partial or total inversion of the viscera, resembling the mirror image of a normal person, is not associated with cardiovascular or visceral developmental malformations. (4) Enlarged lymph nodes in the abdominal cavity and other nodular masses in the abdominal cavity: There was a history of inflammation or malignant tumors, often multiple, located in the abdominal cavity and retroperitoneum, without a splenic hilar incision or splenic hilar vessels, and enhancement showed homogeneous enhancement in all three phases.

4.2.8 *Conclusion*

PS is a complex malformation syndrome involving multiple systems. The most common malformations are short pancreas, and other malformations, such as left lung heterogeneity, hepatic segmental IVC agenesis with continuation of the azygos vein, midgut malrotation, preduodenal portal vein, and left atrial heterotaxy, have relatively high prevalence rates.

# References

1. Abdullah RA, Alharbi MA, Alnutaifi FM, Faqih AI, Alduraibi KM, Alharbi AS. Polysplenia Syndrome Complicated by Splenic Infarction: A Report of a Rare Case. *Cureus.* 2023;15:e38589.
2. Frescura C, Ho SY, Giordano M, Thiene G. Isomerism of the atrial appendages: morphology and terminology. *Cardiovasc Pathol.* 2020;47:107205.
3. Malki MC, Outznit M, Mechhor S, et al. Polysplenia syndrome in adulthood: a case report. *Pan*

*Afr Med J.* 2022;41:67.

4. Rameshbabu CS, Gupta KK, Qasim M, Gupta OP. Heterotaxy Polysplenia Syndrome In An Adult With Unique Vascular Anomalies: Case Report With Review Of Literature. *J Radiol Case Rep.* 2015;9:22-37.
5. Mountassir ME, Borahma M, Benelbarhdadi I, Lagdali N, Hosni A, Ajana FZ. Polysplenia syndrome revealed in adulthood by pancreatic and vascular malformations: a case report. *Pan Afr Med J.* 2022;43:77.
6. Tawfik AM, Batouty NM, Zaky MM, Eladalany MA, Elmokadem AH. Polysplenia syndrome: a review of the relationship with viscero-atrial situs and the spectrum of extra-cardiac anomalies. *Surg. Radiol. Anat.* 2013;35:647-653.
7. Vaughan TJ, Hawkins IF, Jr., Elliott LP. Diagnosis of polysplenia syndrome. *Radiology.*

1971;101:511-518.

8. Gayer G, Apter S, Jonas T, et al. Polysplenia syndrome detected in adulthood: report of eight cases and review of the literature. *Abdom Imaging.* 1999;24:178-184.
9. Peoples WM, Moller JH, Edwards JE. Polysplenia: a review of 146 cases. *Pediatr Cardiol.*

1983;4:129-137.

10. Low JP, Williams D, Chaganti JR. Polysplenia syndrome with agenesis of the dorsal pancreas and preduodenal portal vein presenting with obstructive jaundice--a case report and literature review. *Br J Radiol.* 2011;84:e217-220.
11. Maier M, Wiesner W, Mengiardi B. Annular pancreas and agenesis of the dorsal pancreas in a patient with polysplenia syndrome. *AJR Am J Roentgenol.* 2007;188:W150-153.
12. Kayhan A, Lakadamyali H, Oommen J, Oto A. Polysplenia syndrome accompanied with situs inversus totalis and annular pancreas in an elderly patient. *Clin. Imaging.* 2010;34:472-475.
13. Kobayashi H, Kawamoto S, Tamaki T, Konishi J, Togashi K. Polysplenia associated with semiannular pancreas. *Eur. Radiol.* 2001;11:1639-1641.
14. Ito H, Ohgi S, Kanno T, Ishibashi T. Heterotaxy syndrome with pancreatic malrotation: CT features. *Abdom Imaging.* 2003;28:856-858.
15. Burton EC, Olson M, Rooper L. Defects in laterality with emphasis on heterotaxy syndromes with asplenia and polysplenia: an autopsy case series at a single institution. *Pediatr Dev Pathol.* 2014;17:250-264.
16. Hadar H, Gadoth N, Herskovitz P, Heifetz M. Short pancreas in polysplenia syndrome. *Acta Radiol.* 1991;32:299-301.
17. Herman TE, Siegel MJ. Polysplenia syndrome with congenital short pancreas. *AJR Am J Roentgenol.* 1991;156:799-800.
18. Fulcher AS, Turner MA. Abdominal manifestations of situs anomalies in adults. *Radiographics.*

2002;22:1439-1456.

19. Li ZG, Lin ZC, Mu HY. Polysplenia Syndrome With Splenic and Skeletal Muscle Metastases From Thyroid Carcinoma Evaluated by FDG PET/CT: Case Report and Literature Review: A Care-Compliant Article. *Medicine (Baltimore).* 2016;95:e2532.
20. G AL, Kagali NA, M S, Prasad BS. Preduodenal portal vein in adult with polysplenia syndrome revisited with a case report. *Indian J. Surg.* 2014;76:137-142.
21. Seo HI, Jeon TY, Sim MS, Kim S. Polysplenia syndrome with preduodenal portal vein detected in adults. *World J Gastroenterol.* 2008;14:6418-6420.
22. Salma EH, Najwa A, Mehdi T, Nazik A, Latifa C, Siham EH. Polysplenia Syndrome With

Persistent Left Superior Vena Cava: Case Report and Review of the Literature. *Glob Pediatr Health.* 2022;9:2333794X221127640.

23. Lai B, Zhong J, Zhong J. Polysplenia syndrome complicated with posterior nutcracker syndrome. *Asian J. Surg.* 2023;46:5460-5461.
24. Montero-Macias R, Krief D, Villefranque V, Kilani M, Castillo-Tunon JM. Anatomy, embryology, and imaging of situs ambiguous with polysplenia and left IVC. *Radiol Case Rep.* 2023;18:1727-1732.
25. Yorita K, Sasaki S, Kawada A, et al. Hepatoid Adenocarcinoma of the Extrahepatic Bile Duct in a Patient with Polysplenia Syndrome. *Intern. Med.* 2017;56:401-407.
26. Mimatsu K, Oida T, Kano H, et al. Preduodenal portal vein, intestinal malrotation, polysplenia, and interruption of the inferior vena cava: a review of anatomical anomalies associated with gastric cancer. *Surg Radiol Anat.* 2012;34:179-186.
27. Iwamura T, Shibata N, Haraguchi Y, et al. Synchronous double cancer of the stomach and rectum with situs inversus totalis and polysplenia syndrome. *J. Clin. Gastroenterol.* 2001;33:148-153.
28. Yoneyama H, Kondo C, Yamasaki A, Nakanishi T, Sakai S. Comparison of situs ambiguous patterns between heterotaxy syndromes with polysplenia and asplenia. *Eur. J. Radiol.* 2015;84:2301-2306.

Fig 1. (a) Left lung heterogeneity: Sagittal image of the right lung showing only the major oblique fissure (arrow). (b) A short pancreas (△). (c) Visceral inversion: The liver was located on the left side, the stomach (St) was on the right side, and multiple splenic nodules (PSs) were observed in

the right upper quadrant. (d) Horizontal liver. (e) Hepatic hilar cholangiocarcinoma (arrow) and median gallbladder (GB). (f) Malrotation of the midgut (arrow). (g) Dextrocardia. (h) Left atrial heterotaxy. (i) Dextral pulmonary trunk (DPT); the IVC is on the left, and the descending aorta (DA) is on the right. (j) The thickening AZ (arrow) and absence of the IVC of the hepatic segment. (k) The hepatic segment of the IVC is absent, and the left, middle, and right hepatic veins converge and are injected directly into the right atrium (arrow); a thickened AZ (dotted arrow). (l) Duodenal duodenum anterior portal vein (PDPV) (arrow). (m) The portal vein travels anteriorly to the head of the pancreas and then bifurcates into two branches, with the thicker branch traveling along the anterior edge of the liver to the diaphragmatic surface of the liver to enter the liver (solid arrow) and the thinner branch entering the liver in the first hepatic hilar (dotted arrow). (n) Left renal veins converging directly into the azygos vein (arrow). (o) The left renal vein crosses the abdominal aorta (AA) posteriorly to join the Az (arrow), and the right renal vein joins directly to the Az; malrotation of the midgut. (p) Bilateral common carotid artery shared trunks. Aberrant right subclavian artery.

Table 1. Clinical data of 12 cases with polysplenic syndrome

| Case No. | Age (Year) | Sex | Medical history |
| --- | --- | --- | --- |
| 1 | 70 | F | Polyp of the colon |
| 2 | 38 | M | VSD |
| 3 | 53 | F | N |
| 4 | 38 | F | N |
| 5 | 74 | M | Cholangiocarcinoma, cirrhosis |
| 6 | 69 | F | Inflammation of the bile ducts and duodenum |
| 7 | 64 | F | Gallstones |
| 8 | 66 | M | Cholangiocarcinoma, Cholecystitis |
| 9 | 31 | M | Arrhythmia, right kidney stone |
| 10 | 30 | M | N |
| 11 | 57 | F | Left kidney stone |
| 12 | 65 | F | N |

Table 2. The malformations of 12 cases with polysplenic syndrome on CT

| **Malformations** | **Case1** | **Case2** | **Case3** | **Case4** | **Case5** | **Case6** | **Case7** | **Case8** | **Case9** | **Case10** | **Case11** | **Case 12** |
| --- | --- | --- | --- | --- | --- | --- | --- | --- | --- | --- | --- | --- |
| Left lung heterogeneity | Yes | Yes | Yes | Yes | Yes | No | Yes | Yes | No | No | Yes | Yes |
| Left atrial heterotaxy | Yes | Yes | No | No | Yes | No | Yes | Yes | No | No | No | No |
| Location of spleen | Left | Left | Right | Left | Left | Left | Left | Left | Left | Left | Right | Right |
| Number of spleens | 5 | 12 | 4 | 7 | 4 | 3 | 11 | 5 | 12 | 5 | 8 | 5 |
| Liver: small left lobe | Yes | No | No | No | No | No | No | No | No | No | No | No |
| Left liver | No | No | Yes | No | No | No | No | No | No | No | No | Yes |
| Horizontal liver | No | No | No | No | No | No | Yes | Yes | No | No | Yes | No |
| Location of gallbladder | Right | Right | Left | Right | Right | Right | Right | Median | Right | Right | Right | Left |
| Short pancreas | Yes | Yes | Yes | Yes | Yes | Yes | Yes | Yes | Yes | Yes | Yes | Yes |
| Midgut malrotation | Yes | Yes | Yes | No | Yes | No | Yes | Yes | Yes | No | Yes | No |
| Hepatic segmental IVC agenesis with continuation of AZ | Yes | No | No | Yes | Yes | No | Yes | Yes | Yes | Yes | Yes | Yes |
| Bilateral IVC | No | No | Yes | No | No | No | No | No | No | No | No | No |
| PDPV | No | Yes | No | Yes | Yes | No | No | No | Yes | Yes | Yes | No |
| Double LRV | Yes | No | No | No | No | No | No | No | No | No | No | No |
| Double RRV | No | No | Yes | No | No | No | No | No | No | No | No | No |
| Bilateral RV inflow AZ | No | No | No | No | No | No | No | Yes | No | No | No | No |
| LRV travels behind AA, and bilateral RV flow into AZ | No | No | No | No | No | No | No | No | Yes | No | No | No |
| RRV flows into AZ, LRV flows into HAZ | No | No | No | No | No | No | No | No | No | No | Yes | Yes |
| Bilateral CCA shared trunks | Yes | No | No | No | No | No | No | Yes | No | No | No | No |
| ARSA | Yes | No | No | No | No | No | No | Yes | No | No | No | No |
| RAA | No | No | Yes | No | No | No | No | Yes | No | No | No | No |
| DPT | No | No | Yes | No | No | No | No | Yes | No | No | No | No |
| Dextrocardia | No | No | Yes | No | No | No | No | Yes | No | No | No | No |
| SIT | No | No | Yes | No | No | No | No | No | No | No | No | Yes |
| The anterior branch of the portal vein enters the liver from the anterior edge of the liver | Yes | No | No | No | No | No | No | No | No | No | No | No |

IVC-inferior vena cava; AZ-azygos vein; PDPV-Preduodenal portal vein; RV-renal vein; LRV-left renal vein; RRV- right renal vein; AA-abdominal aorta; SVC-superior vena cava; HAZ-hemiazygos vein; ARSA- aberrant right subclavian arteries; CCA-common carotid arteries; SIT-Situs inversus totalis; RAA-right aortic arch; DPT- dextarl pulmonary trunk
