# Supplementary material for: Analysis of associated malformations by computed tomography in adults with polysplenia syndrome: A pilot study
Source: PLoS One. 2024 Dec 20;19(12):e0312548. doi: 10.1371/journal.pone.0312548 (PMC11661641; doi:10.1371/journal.pone.0312548)
Supplement: Attachment — Submitted filename: point-by-point responses.docx [file pone.0312548.s002.docx]

**Responses to comments:**

We feel great thanks for your professional review work on our article. As you are concerned, there are several problems that need to be addressed. According to your nice suggestions, we have made extensive corrections to our previous draft, the detailed corrections are listed below:

**For editor comments：**

1. When submitting your revision, we need you to address these additional requirements.

Please ensure that your manuscript meets PLOS ONE's style requirements, including those for file naming. The PLOS ONE style templates can be found at

https://journals.plos.org/plosone/s/file?id=wjVg/PLOSOne_formatting_sample_main_body.pdf and

https://journals.plos.org/plosone/s/file?id=ba62/PLOSOne_formatting_sample_title_authors_affiliations.pdf

**Answer：***We had revised my paper according to PLOS ONE's style requirements.*

2. We suggest you thoroughly copyedit your manuscript for language usage, spelling, and grammar. If you do not know anyone who can help you do this, you may wish to consider employing a professional scientific editing service.

The American Journal Experts (AJE) (https://www.aje.com/) is one such service that has extensive experience helping authors meet PLOS guidelines and can provide language editing, translation, manuscript formatting, and figure formatting to ensure your manuscript meets our submission guidelines. Please note that having the manuscript copyedited by AJE or any other editing services does not guarantee selection for peer review or acceptance for publication.

Upon resubmission, please provide the following:

The name of the colleague or the details of the professional service that edited your manuscript

A copy of your manuscript showing your changes by either highlighting them or using track changes (uploaded as a *supporting information* file)

A clean copy of the edited manuscript (uploaded as the new *manuscript* file)

**Answer：***We had employed a professional scientific editing service of AJE.*

3. We note that the grant information you provided in the ‘Funding Information’ and ‘Financial Disclosure’ sections do not match.

When you resubmit, please ensure that you provide the correct grant numbers for the awards you received for your study in the ‘Funding Information’ section.

**Answer：***The funders had no role in study design, data collection and analysis, decision to publish, or preparation of the manuscript. We had added this sentence in Statement.*

4. We note that your Data Availability Statement is currently as follows: All relevant data are within the manuscript and its Supporting Information files.

Please confirm at this time whether or not your submission contains all raw data required to replicate the results of your study. Authors must share the “minimal data set” for their submission. PLOS defines the minimal data set to consist of the data required to replicate all study findings reported in the article, as well as related metadata and methods (https://journals.plos.org/plosone/s/data-availability#loc-minimal-data-set-definition).

For example, authors should submit the following data:

- The values behind the means, standard deviations and other measures reported;

- The values used to build graphs;

- The points extracted from images for analysis.

Authors do not need to submit their entire data set if only a portion of the data was used in the reported study.

If your submission does not contain these data, please either upload them as Supporting Information files or deposit them to a stable, public repository and provide us with the relevant URLs, DOIs, or accession numbers. For a list of recommended repositories, please see https://journals.plos.org/plosone/s/recommended-repositories.

If there are ethical or legal restrictions on sharing a de-identified data set, please explain them in detail (e.g., data contain potentially sensitive information, data are owned by a third-party organization, etc.) and who has imposed them (e.g., an ethics committee). Please also provide contact information for a data access committee, ethics committee, or other institutional body to which data requests may be sent. If data are owned by a third party, please indicate how others may request data access.

**Answer：***We had checked the raw data which had be showed in tables in this paper.*

5. When completing the data availability statement of the submission form, you indicated that you will make your data available on acceptance. We strongly recommend all authors decide on a data sharing plan before acceptance, as the process can be lengthy and hold up publication timelines. Please note that, though access restrictions are acceptable now, your entire data will need to be made freely accessible if your manuscript is accepted for publication. This policy applies to all data except where public deposition would breach compliance with the protocol approved by your research ethics board. If you are unable to adhere to our open data policy, please kindly revise your statement to explain your reasoning and we will seek the editor's input on an exemption. Please be assured that, once you have provided your new statement, the assessment of your exemption will not hold up the peer review process.

**Answer：***We had checked the raw data which had be showed in tables in this paper.*

6. PLOS requires an ORCID iD for the corresponding author in Editorial Manager on papers submitted after December 6th, 2016. Please ensure that you have an ORCID iD and that it is validated in Editorial Manager. To do this, go to ‘Update my Information’ (in the upper left-hand corner of the main menu), and click on the Fetch/Validate link next to the ORCID field. This will take you to the ORCID site and allow you to create a new iD or authenticate a pre-existing iD in Editorial Manager.

**Answer：***We had added the ORCID of corresponding author.*

**For review comments**

**Reviewer1：**

1. Rigorously written article by the authors. It is a retrospective series, however the rarity of the condition makes the article useful. Adding percentages of findings in tables would have added to the readability of the article.

**Answer：***We had added the percentages of findings in table.*

**Reviewer2:**

1. You can shorten the running title as the main title and running title appear to be the same.

**Answer：***We think this is an excellent suggestion. We have simplified the running title to “Malformations in Adults with Polysplenia on CT”.*

2. Keywords may be chosen a bit more appropriately, for example, you may use a keyword like malformations which may be used instead of ＂adult＂ making it a bit more specific and catchy.

**Answer：***Thanks for your suggestion. We have replaced the keyword “adult” with “malformations”.*

3. The sample size being 12, I request you to make a small change in your title if possible, you can call this as a ＂pilot＂ study.

**Answer：***We agree with your suggestion. We have added “A Pilot Study” in Title.*

4. In financial disclosure section, you have disclosed that there was no funding prpvided for your study. Under the fundings head, you are mentioning certain project names. Can you please clarify it further on how the projects helped in your study funding? Was it based on collection of patients or something related?

**Answer：***The funders had no role in study design, data collection and analysis, decision to publish, or preparation of the manuscript. We had added this sentence in Statement.*

5. In your objective, can you replace the word ＂summarise＂ to ＂analytically depict＂ so that it becomes more measurable and oriented to your study results.

**Answer：***As suggested by the reviewer, we have replaced the word “summarise” with “analytically depict”.*

6. If you can have some statistical tests suitaing this quantitative study wherein you can further supplement your results with statisical evidence, it would make your study stronger.

**Answer：***Thank you for the valuable suggestions. We had added the percentages of findings in result and table 2.*

7. Were the examining radiologists blinded from the patients? Were the patients blinded from the examining radiologists? Were both blinded from each other? Were the examining radiologists aware that this sort of study may take place in future subject to their reporting?

**Answer：***The study is a retrospective study. We retrospectively analyzed the CT imaging data of twelve adults of PS to summarize the concomitant deformities in adults of PS with the aim of improving the understanding of concomitant deformities of PS, avoiding misdiagnosis of anatomical variants as lesions and causing unnecessary surgical injuries.*

**Reviewer3:**

1. Kindly add full form of CT in the title.

**Answer:** *Thank you for the valuable suggestions. As suggested by the reviewer, we have added the full form of CT (Computed Tomography) in the title.*

2. Kindly add an ＂s＂after ＂patient＂.

**Answer:** *Thank you for your detailed review. We have made the necessary corrections to the term “patient”.*

3. Kindly correctly label the figure 1b and figure 1c.

**Answer:** *Thanks for this comment. We have corrected the label of the figure 1b and figure 1c.*

4. “observed” would be a better term than “affected”.

**Answer:** *Thanks for your suggestion. We have replaced “affected” with “observed” as suggested.*

5. Throughout the manuscript, kindly use full form of the acronym when it is used as a first word of the sentence.

**Answer:** *As suggested by the reviewer, we have changed the abbreviations at the beginning of paragraphs throughout the manuscript to their full forms.*

6. Kindly use a widely accepted term of enhancement for describing the pattern of enhancement. Something like Zebra pattern.

**Answer:** *We appreciate your suggestion and have made the necessary changes, replacing “blotchy” enhancement with “Zebra pattern” enhancement.*

7. Pancreas would suffice.

**Answer:** *Your suggestion is greatly appreciated. We have made the change “Pancreases” to “Pancreas”.*

8. Rewrite the sentence ＂Since the normal pancreas...＂ for clarity.

**Answer:** *We sincerely thank the reviewer for careful reading. As suggested by the reviewer, we have revised the sentence to “The pancreas is formed by the fusion of ventral and dorsal pancreatic buds; the ventral pancreatic buds give rise to the uncinate process and head, and the dorsal pancreatic buds give rise to the body and tail [14]. When the pancreas is dorsally underdeveloped, dysplasia of the body and tail of the pancreas, occurs which mostly manifests as a short pancreas [14].”.*

9. Kandly identify the site of gall bladder.

**Answer:** *Thank you for your suggestion. We had checked the site of gall bladder.*

10. This statement contradicts the statement in the results section and image 1m. Kindly confirm and rewrite.

Answer: *We had made a consistent statement for this finding in our paper.*

11. Conclusion should be a separate heading and not a subheading. Kindly rectify.

**Answer:** *As suggested by the reviewer, we have revised the heading of Conclusion.*

12. Kindly elaborate what does the white arrow in the part (d) indicates.

**Answer:** *It showed the hirizontal liver and biliary stones. To avoid misunderstanding, we had deleted this arrow.*

13. Kindly enlarge the dotted arrow in the figure 1k and figure 1m.

**Answer:** *We had enlarged the dotted arrows in the fig 1k and fig 1m.*

14. Kindly add arrows to the image 1p.

**Answer:** *We had added the arrows for image 1p.*

15. Kindly link the table 2 in the text.

**Answer:** *We had linked the table 2 in the text.*

**Reviewer4:**

1. With 12 cases, the topic should be better under a case review series rather than a research article.

**Answer:** *Thank you for the helpful suggestion. We had added the A PILOT STUDY in Title.*

2. Author/s should describe the spectrum of heterotaxy or situs ambiguus syndromes in a small paragraph in the discussion introduction.

Answer: *We had added some sentences about heterotaxy or situs ambiguous in the first paragraph in discussion.*

3. The author/s should mention in a concise paragraph the need for symptomatic management in the patients, depending on the type of associated anomaly, along with the importance of frequent follow-up with a multidisciplinary team.

**Answer:** *We had added a concise paragraph, “Being familiar with the occurrence of malformations associated with PS is conducive to avoiding misdiagnosis and mistreatment. Because the malformations involve multiple systems, multidisciplinary consultation is more conducive to the evaluation of this disease.”, as last paragraph.*

4.Fig 1l Duodenal duodenum anterior portal vein (PDPV) - correct the terminology

**Answer:** *Thank you for your suggestion. We have corrected ＂Duodenal duodenum anterior portal vein (PDPV)＂ to ＂Preduodenal portal vein (PDPV)＂.*
